# Supplementary material for: Guidelines for a participatory Smart City model to address Amazon’s urban environmental problems
Source: PeerJ Comput Sci. 2023 Dec 12;9:e1694. doi: 10.7717/peerj-cs.1694 (PMC10773765; doi:10.7717/peerj-cs.1694)
Supplement: Supplemental Information 1 [file peerj-cs-09-1694-s001.pdf]

# Appendix 1 – Questionnaire to consult Manaus´s City Hall Managers

1) Vision: What is the long-term vision and strategy to transform Manaus into a Smart City?

2) Vision: Where can citizens find the Strategic Plan or Project to achieve this vision?

3) Leadership: Who are the leaders responsible for managing this Plan/Project?

4) Budget: What is the budget allocated for this undertaking?

5) Budget: In which official gazette was the budget published

6) Financial Incentives: What financial incentives is the City offering to encourage entrepreneurs, universities, startups, and organizations to support the Smart City project?

7) Support Programs: What programs has the city developed to encourage private sector participation?

8) Talent Preparation: What plan was created to educate citizens and provide skills needed for a Smart City?

9) Citizen Participation: What initiatives engaged citizens in the Strategic Plan/Project?

10) Citizen Participation: How will the city guarantee Smart City benefits reach most citizens?

11) Innovation Ecosystem: Who are the local innovation partners involved and what are their responsibilities?

12) Innovation Ecosystem: What are the partners' responsibilities?

13) Smart Policies: What public policies were effectively developed to make Manaus Smart?

14) Smart Policies: What actions updated regulations to support this undertaking?

15) Historical Record: Which good practices have been recorded?

16) Which Smart City model is being implemented?

17) Where can citizens access this information on the Manaus City Hall website?
