# Supplementary material for: Guidelines for a participatory Smart City model to address Amazon’s urban environmental problems
Source: PeerJ Comput Sci. 2023 Dec 12;9:e1694. doi: 10.7717/peerj-cs.1694 (PMC10773765; doi:10.7717/peerj-cs.1694)
Supplement: Supplemental Information 2 [file peerj-cs-09-1694-s002.pdf]

Appendix 2 – Questionnaire formulated to consult Manaus´s citizens.

Goals: 1) Assess respondent's knowledge and interest in Smart Cities, Decarbonized Cities, and Disruptive Technologies; 2) Identify perceptions of key environmental issues in Manaus.

Q1) Rate your knowledge level on the following: Smart Cities, Decarbonized Cities, Disruptive Technologies (AI, IoT, Blockchain, etc.).

Choices: None, Low, Medium, High

Q2) Since 2016, when a mayoral candidate promised to make Manaus a Smart City, how many times have you been consulted to help with this project?

Choices: 0, 1, 2, 3, More than 3

Q3) As a citizen, how many times since 1988 have you been invited by government to develop sustainable plans for the State of Amazonas or Manaus?

Choices for Federal, State, and Municipal governments: 0, 1, 2, 3, 4, More than 4

Q4) Would you accept an invitation to help develop a long-term plan to make Manaus a Smart City?

Choices: Yes, No, Maybe

Q5) In which area would you like to help?

Choices: None, Social Services, Culture, Education, Environment, Sports, Finance, Governance, Transportation, Housing, Health, Infrastructure, Other

Q6) What are the top 5 environmental issues affecting you in Manaus?

Choices: Insufficient urban trees/greenery, Increased air pollution, Increased noise pollution, Polluted rivers, Garbage accumulation, Flooding, Rising temperatures, Traffic congestion, Visual pollution, Green space destruction, Water waste, Food waste, Energy waste, Public space invasion, Gas emitting vehicles, Urban fires, Other

Q7) Would you help develop a plan to decarbonize Amazon's economy by 2050 if invited?

Choices: Yes, No, Maybe

Q8) What education would you like to receive on these topics?

Choices: Short course on Smart/Decarbonized Cities, Basic disruptive tech course, Advanced disruptive tech course, Smart City certificate, Smart City Master’s, Smart City PhD, Other

Q9) What is your highest education level?

Choices: Primary school, Secondary school, Undergraduate, Postgraduate certificate, Master’s, PhD, Other

Q10) What is your age group?

Choices: Under 18, 18-24, 25-30, 31-40, 41-50, Over 50

Q11) What is your gender? Choices: Male, Female, Prefer not to say, Other

Q12) What neighborhood do you live in? Open response

Q13) Please share any suggestions. Open response
