# Supplementary material for: Guidelines for a participatory Smart City model to address Amazon’s urban environmental problems
Source: PeerJ Comput Sci. 2023 Dec 12;9:e1694. doi: 10.7717/peerj-cs.1694 (PMC10773765; doi:10.7717/peerj-cs.1694)
Supplement: Supplemental Information 4 [file peerj-cs-09-1694-s004.pdf]

Appendix 4 – Table A1 Example of Enablers that support Smart Cities

Enablers	Description
National Policies, Programs and Projects that improved ICT infrastructure, regulations, as well as modernized the State toward e-Government and Digital Society.	From 1986, Singapore National IT Plan, followed by Singapore One (nationwide broadband network), started in 1997, Wireless@SG Program developed in 2006 to offer free wi-fi island-wide.<br>From 2015 Singapore eGov2015 Master Plan (Singapore Government, 2015).
	The Telecities Network Project launched in 1993 by the EU (Götzl, 2002) benefited more than 120 European cities.
	From 1994 Japan´s Master Plan to Promote Government-wide use of IT. From 2001, it created the IT Strategic Headquarters and e-Japan Strategy (Kaneko, 2001), followed by 2004 with the u-Japan Policy Package, and from 2006, with the u-Japan Policy Promotion Program.
	From 1995, China plans Eight Gold (9th Five Year), followed by Digital Cities (from 1998; 10th Five Year), and Information Cities Plan (from 2006; 11th Five Year) (Wang, Zhang, and Zhong, 2019).
	South Korea, in 2009, launched the Comprehensive National Ubiquitous City Plan 1. After review, in 2013, it was updated to National Ubiquitous City Comprehensive Plan 2.
National Policies, Projects, Public Call or Challenge to foster Intelligent or Smart Cities (including Pilot Test)	Since 1998, the Chinese government has created several plans: National Digital City, National Smart City Policies, New Smart Cities, followed by National Smart City Pilot (Atha, et al. 2020 p. 14-15; Shan et al., 2021; Wang, Zhang, and Zhong, 2019; Wang, Loo, and Huang, 2021).
	In 2005, Intelcities (Intelligent Cities) Project was launched (European Commission, 2005) to support EU policy goal towards Knowledge Society by 2010, which benefited more than 20 European Cities. The Smart City and Communities Initiative launched in 2011 (Kylili and Fokaides, 2015; European Commission, 2011).<br>In 2017 and 2020, the EU launched the Digital City Challenge (European Commission, 2017; Living in Europe, 2020), which benefited 115 cities in the EU.
	In 2008, in the USA, IBM started the Smarter Planet Strategy Agenda.<br>In 2009, IBM launched the Smarter City Campaign.<br>In 2010, IBM Smart City Challenge (IBM, 2017) helped 114 cities around the world until May 2014 (Sogeti Labs, 2014).

Continuation: National Policies, Projects, Public Call or Challenge to foster Intelligent or Smart Cities (including Pilot Test)	In 2015, Obama announced New Smart Cities Investments (White House, 2015), and in 2016, the US Department of Transportation launched Smart City Challenge, a public call that selected seven cities to implement smart projects to improve transportation systems (US Department of Transportation, 2017).
	In 2014, Singapore launched the Smart Nation Program Office & Smart Nation Initiative (Singapore Government, 2015).<br>In the same year, Finland created the Six Cities Strategy (6Aika, 2014)
	In 2017, SK developed the National Smart City in Sejong and Busan.<br>In 2018, they launched Smart City Innovation Growth Engine R&D with a budget of 1,313 billion won for the period of 2018 to 2022, benefiting Daegu Metropolitan City and Gyeonggi-do city.<br>In 2019, the National Smart City Challenge begins (South Korea Government Ministry of Land, Infrastructure and Transport, 2020).
Alliances, Budgets, Incentives, Financial or/and Technical Support, Annual Events	Since 1995, the World Bank Group has started to finance Digital Government Projects – GovTech Agenda. As a result, until October 2022, 1065 projects have already been implemented, 314 are operational, and 70 are in the pipeline, resulting in a portfolio with 1449 large ICT/e-Gov components projects funded in 147 countries (WBG, 2020; 2022).<br>In 2018, created the Global Smart City Partnership Program, to help WBG clients and teams to make the best use of technologies, data, and available resources, contributing to improve city planning, management, service delivery, citizen engagement, improve government accountability, knowledge-sharing and dissemination through the Smart Cities Knowledge Silo Breaker. (WBG, 2018).
	In 2009, Amsterdam Smart City was initiated as a collaboration between the Liander Grid Operator and Amsterdam Innovation Motor (AIM), which involved a close connection with the municipality of Amsterdam and citizens (Capra, 2014 p. 40).
	Since 2011, Spain has organized the Spain Barcelona SC Expo World Congress, and Barcelona City implemented the Smart City Strategy with alliances between public and private sectors like CISCO, IBM, Philips, SAP, Schneider and GDF Suez/ Engie, i2CAT, CESCA, the Dublin Institute of Technology, business schools, etc. (Ferrer, 2017 p.72)
	Since 2010, Japan METI has supported the Yokohama Smart City Project as part of the Next Generation Energy and Social System Demonstration Area Program (City of Yokohama, 2015) based on Public-Private Partnership,

Continuation:<br><br>Alliances, Budgets, Incentives, Financial or/and Technical Support, Annual Events,	involving 24 organizations such as TEPCO, Tokyo Gas, Toshiba, Taisei, Meiden, Azbil, SHMZ, NTT, Mobility Power, Panasonic, ORIX, Nissan, etc.<br>Since 2012, Japan has organized the Asian Smart City Conference in Yokohama (ASCC, 2012).
	In May 2016, inspired by SDG 11, The United Nations Economic Commission for Europe (UNECE) and the International Telecommunication Union (ITU) with several partners launched The United for Smart Sustainable Cities (U4SSC) to encourage organizations to use ICTs for the transition to Smart Sustainable Cities (UNECE, 2016).<br>In 2019, the G20 created the Global Smart City Alliance (World Economic Forum, n.d.).<br>In the end of 2021 and in the beginning of 2022, the EU launched two ambitious public calls that selected 100 EU cities and 12 additional cites from associated countries, to be part of the EU Mission for 100 Climate Neutral and Smart Cities by 2030, with €360 million from Horizon Europe funding, covering the period 2022-2023, to start the innovation ways towards Netzero by 2030 (European Commission, 2021; 2022).
