# Supplementary material for: Guidelines for a participatory Smart City model to address Amazon’s urban environmental problems
Source: PeerJ Comput Sci. 2023 Dec 12;9:e1694. doi: 10.7717/peerj-cs.1694 (PMC10773765; doi:10.7717/peerj-cs.1694)
Supplement: Supplemental Information 5 [file peerj-cs-09-1694-s005.pdf]

Appendix 5 – Table A2 Profile of Twenty-Five Benchmark Smart Cities

Rank and Initiatives	Smart City Definition (D), Type (T), and Vision(V)	Terms and Digital Platforms	Fab, Living or Urban Labs
**First place**<br><br>**Amsterdam (NL)**<br>In 1994: Virtual Digital City (De Digital Stad-DDS)<br><br>Since 2009: Amsterdam Smart City Program<br>In 2015: IBM Smart City Challenge (SCC) Winner<br>Since 2019: Amsterdam Climate Neutral2050 RoadMap Local Government and Private Sector	D: Not found.<br><br>Phase 1: 2009 – 2011 focused on **energy efficiency**. Phase 2: 2012 – 2014 focused on **energy efficiency** and **internet-related applications** (Capra 2014, p. 40).<br><br>V1: In 2020, Amsterdam will be one of the **most sustainable cities** in the world (Osieck, 2011 p. 155)<br><br>T: **Open Innovation Platform**.<br><br>V2: An **open innovation platform** that brings together **innovation** professionals from **government, companies, knowledge institutions, and civil society organizations** to shape the **city** and region of the future. (Amsterdam Smart City, 2022)	Fields: Economy, Environment, Living, Governance, People<br><br>Goal: Efficiency, QoL, Sustainability<br>TA: Technology<br>Other: Apps, Open Innovation, Stakeholders<br><br>Digital Platforms to Engage Citizens: Amsterdam Smart City Platform, Amsterdam Participatie, Amsterdam Smart Citizens Lab, CityData, Smart Health Amsterdam site, Budgetmonitoring, Amsterdam Smart City Living Lab, GVB app, LEAP, MyCleanCity app, Amsterdam Data Platform data.amsterdam.nl/	Amsterdam Energy City Lab<br><br>Circle Lab<br><br>Amsterdam Smart Citizens Lab<br><br>FabLab Amsterdam<br><br>Living Lab Circular Buiksloterham,<br><br>Living Lab Sloterdijk III<br><br>Waag Research Labs

Rank and Initiatives	Smart City Definition (D), Type (T), and Vision(V)	Terms and Digital Platforms	Fab, Living or Urban Labs
**Second place Singapore**<br>1986: National IT Plan<br>2006: Wireless@SG<br>2011: eGov2015 Master Plan<br>2012: Jurong Lake District won IBM SCC<br>Since 2014: Smart Nation Initiative<br>National, Local, Private	D**:** Not found, but it is pioneering in the concept of Smart Nation (Quélin and Smadja, 2021 p. 18)<br><br>**T: Digital Society**, **Economy**, and **Government**.<br>V: Smart Nation is a transformed Singapore where **people** will be more empowered to **live** meaningful and fulfilled **lives, seamlessly** enabled by **technology**, that offers exciting opportunities for all. It is where **businesses** can be more productive and take advantage of new opportunities in the **digital economy**. (Singapore Government, 2018)	Fields: Economy, People, Living, Governance<br>Goal: Efficiency, QoL<br>TA: Technology<br>Others: Business, Digital Economy, Society, Government, Collaboration<br>Digital Platforms to Engage Citizens:<br>Smart Nation Platform, My Info, SingPass, Health Hub, Hello Lump Posts, SGSecure App, eCitizen, OneService App, CityLab<br>Singapore Data Gov<br>https://data.gov.sg/	Sustainable Living Lab (SL2)<br><br>MPA Living Lab<br><br>PSA Living Lab<br><br>Jurong Port Living Lab<br><br>NTU Smart Nation Translation Lab
**Third Place London (UK)**<br>Since 2013: Smarter London Plan<br><br>Won the ODI Annual Open Data Award 2015<br><br>Since 2018: Smarter London Together Roadmap	D: The general term used to discuss how cities across the globe are **sharing information** and making use of **technology** to work more **efficiently** (Greater London Authority, 2013 p. 58).<br>T1: **Smart**<br>V1: A city that uses the creative power of **data** and **new technologies** to serve London and improve **Londoners' lives** (Greater London Authority, 2013 p. 13; 2016 p. 10)<br>T2: **Collaborative**, **Connected,** and **Responsive city**.	Fields: Economy, Env., Gov., Living, People<br>Goal: Efficiency, Sustainability, QoL<br>TA: Connection, Technology, Information<br>Others: Collaborative, Responsive, Data, City, Digital, Resilient<br>Digital platforms: Talk London, Citymapper, Report It, London Living Streets, City Lab, Live Park, Citizen Lab, Safe & The City App,	CitizenLab<br><br>Smart Mobility Living Lab (SMLL)<br><br>Energy Systems Catapult Living Lab<br><br>University of

Local, Private, National	V2: Integrates **digital technologies** and uses **city-wide data** to the needs of respond to our **citizens** (Greater London Authority, 2018)	London Datastore data.london.gov.uk/ Transport For London (TfL) Open Data http://bit.ly/3EMB7SJ	Greenwich Living Lab
**Fourth place New York (USA)** <br>Since 2007: PlanNYC <br>Since 2011: Digital City Roadmap NYP <br>Since 2015: OneNYC <br>Since 2022: OneNYC 2050 <br>OTI Strategic Plan Local initiative	D1: Any **local government** that leverages **technology** solutions to promote public health, safety and welfare, and improves **quality of life** for the **residents** of the **municipality**, especially traditionally underserved and under-resourced communities (NY State, 2020). D2: A **local government** that uses **technology** and **planning practices** to generate better outcomes for its **residents** through the use of **data**. (NYC (2021) T1: Digital City; V1: be recognized as the world's top-ranked **Digital City**, based on indices of **Internet** access, **Open Government**, **citizen engagement**, and **digital industry** growth (NYC, 2011) T2: Connected City; V2: Ensure **all New Yorkers** have access to effectively engage with the **digital economy** and **society** (NYC OTI, 2022 p. 10)	Fields: Economy, Environment, Governance, Living, People Goal: Efficiency, Sustainability, QoL TA: Connection, Technology Others: Data, Digital City, Digital Industry, Digital Economy, Open Government. Digital platforms: NYC Engage, NYC Open Data, NYC 311, Participatory Budgeting, Urban Innovation Lab, Cowlines App, Nextdoor App, Mytown App, NYC Open Data https://opendata. cityofnewyork.us/data/	NYCx Co-Lab <br>Urban Future Lab <br>Urban Innovation Lab

**Rank and Initiatives**	**Smart City Definition (D), Type (T), and Vision(V)**	**Terms and Digital Platforms**	**Fab, Living or Urban Labs**
**Fifth place Helsinki (Fi)**<br><br>In 2010: Helsinki Region Infoshare Program<br><br>In 2011: Won IBM SCC: A project to open data to citizens.<br><br>Since 2012 (?) Helsink Smart Region<br><br>Since 2014: Smart & Clean Helsinki Metropolitan Area<br>Since 2017: Helsinki City Strategy<br>Local, Private, and Region	Not found Smart City definition.<br>T1: **Living Lab (a platform** for collaborative projects that bring users with their ideas, experiences, and enable the **business community** to develop the products and services that are of the greatest interest to the public) (Hielkema and Hongisto, 2012).<br>V1: Not clear<br>T2: **Platform** for **Smart** and **Clean** Solutions.<br>V2: Turn the Helsinki Metropolitan area into an internationally important reference area for **ecological** and **smart** solutions by 2021 and showcase the **best products and services** of Finnish and international companies. (SITRA, 2015)<br>T3: **Innovation ecosystem** for **Green** and **Digital** Transition.<br>V3: Until 2021, to be the world's most **functional** city. In addition, Helsinki aims to be the city in the world that makes the best use of **Digitalization** (Helsinki, 2017 p. 16).<br>V4: Not clear but focuses on three themes until 2030: **Citizens´s City, Climate Neutrality, and Industrial Modernization**. (Helsink Smart Region, 2022)	Fields: Environment, Governance, Living, People, Economy<br><br>Goal: Efficiency, Sustainability, QoL<br><br>TA: Technology<br><br>Others: Data, Living Lab, Platform, Innovation, City, Industrial Modernization, Digital, Ecosystem<br><br>Digital Platforms to Engage Citizens:<br>Helsinki Smart Region website<br>Helsinki Regional Infoshare<br>Forum Virium Helsinki<br>Digital Forms<br>Waste Management System<br>FixMyStreet<br>mySMARTLife<br>Open Data Service<br>https://hri.fi/en_gb/	Forum Virium Helsinki<br><br>Kalasatama Urban Lab<br><br>Jatkasaari Smart Mobility Lab<br><br>Helsinki Living Lab<br><br>Mobility Lab Helsinki

Rank and Initiatives	Smart City Definition (D), Type (T), and Vision(V)	Terms and Digital Platforms	Fab, Living or Urban Labs
**6th) Seoul (SK)**<br><br>Since 2011: Smart Seoul 2015<br><br>Since 2016: Global Digital Seoul 2020 Master Plan<br><br>Since 2018: Smart City Strategic Plan<br><br>In 2019: Magok Smart City Living Lab<br><br>Since 2021: Smart Seoul Smart City & Digitalization Master Plan<br>Local, Private, National	Not found local definition, but national<br>D1: A **platform** to improve the **quality of life** of **citizens**, enhance the **sustainability** of cities, and foster **new industries** by utilizing **innovative technologies** of the **fourth industrial revolution** era. (South Korea Ministry of Land, Infrastructure and Transport, 2019 p. 5)<br>D2: A **sustainable city** where various city services are provided based on city **infrastructure** constructed by converging and integrating construction **technologies, information and communications technologies**, etc. to enhance its **competitiveness** and **livability** (South Korea MOLIT, 2021).<br>V1: By 2015, Seoul will become a city that best applies Smart **Technologies**, thought which we will make real your slogan, 'Seoul, a city of happy **citizens** and a city beloved by the world' (Seoul Metropolitan City, 2011 p. 2)<br>T1: **Smart Government**<br>V2: By 2020, a **digital** Seoul made by **citizens** for better **economy** lives to become a **global digital** leader (Seoul Metropolitan Government, 2016 p. 5)<br>T2: Connected, Digital	Fields: Economy, Environment, People, Living<br><br>Goal: Quality of Life, Sustainability<br><br>TA: Technology, ICT,<br><br>Others: Digital, Innovation, Platform, Global<br><br>Digital Platforms to Engage Citizens:<br><br>Seoul Smart City Platform<br><br>Safety e-Report<br><br>Mvoting App and Site<br><br>Open Data Seoul https://data.seoul.go.kr/	Magok Smart City Living Lab (MLL)<br><br>Living Lab in Seoul Innovation Park<br><br>Seongdaegol Living Lab<br><br>Yonsei University´s Information System Lab

	V3: Seoul, Leading the Future of **Digital** Transformation as a **Global Smart City** (Seoul Metropolitan Government, 2021)		
**7th) Copenhagen (DK)**<br><br>In 2012:<br>CPH 2025 climate plan<br><br>In 2013:<br>Won IBM SCC Denmark Digital Growth<br><br>Since 2013:<br>Copenhagen Connecting<br><br>Greater Copenhagen Smart City (?)<br><br>Local & Private	D1: not found, but the smart city initiatives focused on **environmental** issues that are related to the commitment to **carbon neutrality** (Quélin and Smadja, 2021 p. 14)<br>T1: **Carbon Neutral**<br>V1: Make the city **carbon neutral** by 2025, and in doing creating a **greener**, more **sustainable**, and more **livable** capital city, as well as supporting growth (C40 Knowledge, 2017; Quélin and Smadja, 2021).<br>D2: not found; T2: Connected; V2: Be the best **urban environment** and a unique **urban life**.<br>Goals related to Green and Blue Capital, World best city for cyclists, carbon neutral capital, clean and healthy city. Almanac (n.d.); Holm Carlsen (2014).<br>D3: A **living laboratory** for testing smart **technologies** to address the challenges of **urbanization** and **climate change**. Unique access to **data** and **efficient public-private** sector **partnerships** to attract many multinationals. (Copenhagen Capacity, n.d.) T3: **Living Lab** V3: not found.	Fields: Environment, Governance, Living, People<br><br>Goal: Efficiency, Sustainability, Quality of Life<br><br>TA: Technology, Connection<br><br>Other: Living Laboratory, Data, PPP, Stakeholders<br><br>Digital Platforms to Engage Citizens:<br><br>Copenhagen Solutions Lab<br><br>Copenhagen Citizen Service<br><br>Copenhagen City Data Exchange<br><br>WeSolve Better Together site and App<br><br>Open Data DK<br>Opendata.dk/<br>City-of-copenhagen	Copenhagen Solutions Lab<br><br>Copenhagen Living Lab<br><br>Copenhagen Street Lab<br><br>DOLL Living Lab<br><br>EnergyLab Nordhavn<br><br>Energy & Water - Greater Copenhagen Living Lab<br><br>Living Lab Strandvejen

**Rank and Initiatives**	**Smart City Definition (D), Type (T), and Vision(V)**	**Terms and Digital Platforms**	**Fab, Living or Urban Labs**
**8th) Oslo (NO)** (?)<br><br>Not found organized information	D: An **urban area** that has become more **efficient** and/or more **environmentally** friendly and/or more socially **inclusive** using **digital technologies** (Nordic Smart City Network, 2021 p. 6)<br>T: **Open, Connected, Sustainable, and Innovative**<br>V: It is **an urban development vision** to improve the **lives of citizens** by being **open, connected, sustainable, and innovative**. (Nordic Smart City Network, n.d.)	Fields: Economy, Environment, Living, People.<br>Goal: Efficiency, Sust., QoL<br>TA: Connection, Tec.<br>Others: Open, Innovation, Digital.<br>Digital platforms: Futurebuilt, Urban Sharing, Oslo Toll Ring<br>Smart Oslo Accelerator<br>Oslo Statistics Bank<br>statistikkbanken.oslo.kommune.no/webview/	Living Lab Vollebekk<br><br>Oslo Living Lab<br><br>Smart Security Lab
**9th) Vienna (AT)**<br><br>Since 2011: The Big Smart City Wien Initiative<br><br>Since 2014: Smart City Wien Framework Strategy<br><br>Reviewed 2022: Smart Climate City Strategy Vienna<br>Local	D1: A **city** that faces the challenges in the wake of decreasing **resource** consumption combined with rising **demands** (Stadt Wien, 2015 p. 6).<br>T1: **Livable** City; V1: In 2050, Vienna is a **vibrant metropolis** and one of Europe's most **attractive cities**. Goals: To offer optimum **quality of life**, combined with the highest possible preservation of resources, to all **citizens**. This can be achieved through comprehensive **innovations**. (Stadt Wien, 2015 p. 16 and p. 19).<br>D2: The smart city never loses sight of the "**human dimension**"; a city that places the focus on the needs of the **local people**. The development of a	Fields: Environment, Living, People<br><br>Goal: Sustainability, QoL<br><br>TA: Technologies<br>Others: Resource Conservation, Social Innovation, Technical Innovation<br><br>Digital platforms:<br>The Smart City Wien websi+B24te<br><br>junges.wien.gv.at	Aspern Smart City Living Lab<br><br>Siemens Living Lab Process Industries<br><br>Smart City Lab

	**sustainable and livable city** is only successful if everyone benefits, and everyone is able to play their part.<br>T2: **Sustainable, Livable** City<br>V2: a city where **life** is good, but not at the **expense** of the **environment** and thus of **future generations**. Vienna uses the opportunities that **social innovations** and **new technologies** bring with them actively and prudently to achieve the goals. (Stadt Wien, 2020; 2022p 21)	ALBin Project<br><br>Digital School project<br><br>The Vienna City Bike System<br><br>MA 48 Waste App<br>Open Government Data (OGD) Wien Platform<br>digitales.wien.gv.at/ open-data/	
**10th)**<br>**Washington, D.C.**<br>**(USA)**<br><br>Since 2016:<br>Smart DC Initiative<br><br>National	D: One in which **information and communication technology** facilitates improved insight into and control over the various **systems** that affect the **lives of residents**. (Cuddy et al., 2014).<br>T: **Connected** City<br>V: A **city** to **connect** its **communities**, build **economic** strength, protect the natural **environment**, and maintain the safety and security of its **infrastructure.**<br>Goal**:** to become a **connected** city that benefits all components of the **transportation systems–agencies, users**, and infrastructure–and that the transportation systems support the district' systems and broader goals (Washington District Department of Transportation, 2016 p. 1 and p. 25)	Fields: Economy, Environment, Mobility, People.<br><br>Goal: Efficiency, Sustainability<br><br>TA: Connection, ICT<br>Other: Infrastructure, Transportation System<br><br>Digital Platforms to Engage Citizens:<br>OCTO website<br>311DC App<br>My DC Water App<br>Washington Open Data<br>https://data.wa.gov/	The Lab @ DC<br><br>UrbanPlan<br><br>Virginia Tech Urban Living Lab

Rank and Initiatives	Smart City Definition (D), Type (T), and Vision(V)	Terms and Digital Platforms	Fab, Living or Urban Labs
**11th) Zurich (ZH)**<br><br>Since 2018: Strategic Smart City Zürich<br><br>Local Initiative	D: A city that **connects people, organizations,** and **infrastructure** in such a way that **social, ecological, and economic** added value is created. The **networking** of **data, sensors, and applications** allows new and more **efficient solutions** for the **users** of urban infrastructures, as well as for those who operate them. Good **networking** with the **population** strengthens opportunities for **participation** and contact with the **administration** (Zurich City, 2018 p. 4)<br><br>T: **Connected, Collaborative, and Digital** City<br>V: Not found.	Fields: Economy, Env., Governance, Living, People<br>Goal: Efficiency, Sustainability, QoL<br>TA: Connection, ICT, Technology<br>Other: Applications, Data, Stakeholders, Networking, Sensor, Collaborative<br><br>Digital platforms:<br>The Smart City Zurich website, City Lab, The Zurich Pedestrian Navigation System, Pikmi on-demand transport service, ZüriMobil app, My account,<br>Zurich Open Data<br>data.stadt-zuerich.ch/	Reallabor Hunziker Areal<br><br>Hunziker Areal Living Lab<br><br>Smart City Lab
**12th) Berlin (DE)**<br>Since 2015: Smart City Strategy Berlin<br>2017: Launch of Smart City Charter: Making Digital Transformation at the local level sustainable	D1**: Cities** which achieve a significantly higher or stable **quality of life** while using the same or a lower level of **resources**. This can only be achieved by means of an **urban management** which, by using **innovative information and communication technologies**…<br>T1: Smart Administration, E-Government	Fields: Economy, Environment, Governance, Living, People<br>Goal: Efficiency, Sustainability, QoL<br>TA: Connection, ICT, Technology<br>Other: Digital, Integration, Network, Resources, Urban Management, Innovation, Cooperative, Resilient, Open,	CityLAB Berlin,<br><br>Citizens Create Climate Knowledge<br><br>Climate Art Lab<br><br>EUREF Campus<br><br>My-Co Place

2021 Strategic framework Smart City Berlin<br><br>Since 2022: Digital Together: Berlin Strategy<br><br>UE, Private and Local Initiatives	**V1 (?): Smart Berlin is b**oth a place to **live** and an **economic** area which develops **sustainably** through the systematic and **intermodal** deployment of **innovative technologies**, **materials,** and services… (Berlin Senate Department for Urban Development and the Environment, 2015 p. 5)<br>D2: Smart city is **Livable, Lovable, Diverse, Open, Participatory, Inclusive, Climate neutral, Resource efficient, Competitive, Open-Minded, Innovative, Responsive, Sensitive, Safe, and freedom enhancing** city. (BBSR and BMUB, 2017 p. 25)<br>D3: "smart" means tackling challenges **sustainably**, with a focus on the common good and working **collaboratively,** and all the while shaping change processes in a **resilient** manner.<br>T2: **Livable, Digital**<br>V2: A **sustainable, community-oriented, resilient and cooperative** city (Berlin State, n.d; 2022)	Inclusive<br><br>Digital Platforms to Engage Citizens:<br><br>Berlin.de<br><br>Berlin App<br><br>Berlin Citizen Participation Platform<br><br>Berlin Living Lab<br><br>Open Data Berlin https://daten.berlin.de/	SAI-Lab

**Rank and Initiatives**	**Smart City Definition (D), Type (T), and Vision(V)**	**Terms and Digital Platforms**	**Fab, Living or Urban Labs**
**13th) Sydney (AU)**<br><br>Since 2020: Smart City Strategy Framework<br><br>Digital strategy<br><br>Local Initiative	D: Is one that uses **information** and **communications technology** to enhance its **livability, workability, and sustainability**. (City of Sydney, 2020 p. 55; Bane, 2022)<br>T1: **Dynamic, Digital, Responsive**<br>V1: Its vision is a **dynamic, responsive city**, harnessing **technology** and **data** to enable **collaborative innovation** and create a thriving, **inclusive,** and **resilient** future for all. (City of Sydney, 2020a p. 12)<br>T2: **Innovative, Livable**<br>V2: Sydney to continue to be one of the most **innovative** and **livable** cities in the world. (City of Sydney, 2020b)	Fields: Governance, Living, Economy, Environment<br>Goal: Sustainability QoL<br>TA: ICT. Technology<br>Other: Data, Collaborative, Resilient, Innovation, Work<br><br>Digital platforms:<br>Sydney Your Say website, City of Sydney Data hub, The City of Sydney App, cityofsydney.nsw.gov.au<br>City of Sydney Data Hub data.cityofsydney.nsw. gov.au	Intergener8 Living Lab<br><br>Infosys Sydney Living Lab<br><br>Sydney Science Park Living CSIRO Urban Living Lab
**14th) Taipei (Taiwan)**<br>In 2008: The Institute for Information Policy established Living Lab<br><br>In 2013: Won IBM SCC<br><br>Since 2016: Taipei Smart City	D: **Government** as a **Platform**; **City** as a **Living Lab,** focusing on one core vision **(Livable+Sustainable)** plus seven fields: Smart **Security, Building, Transportation, Education, Health, Environment, and Economy**. (Taipei City Government, n.d.)<br>T: **Platform, Living Lab**<br>V: As part of National Vision: Smart **technologies** are applied to solve the pain points of **local government**	Fields: Economy, Environment, Governance, Living, Mobility, People.<br>Goal: Efficiency, Sustainability, QoL<br>TA: Information, Technology<br>Other: Innovation, Stakeholders (PPP), Security, Living Lab, Platform<br>Digital platforms:<br>Living Lab, Tapei Navi, Taipei CooC-Cloud, The Taipei Smart Parking System, Smart Taipei	Fablab Dynamic<br><br>Taipei Smart City Living Lab<br><br>JCA Living Lab

Project Management Office<br>Since: 2020<br>Taipei Smart City 1+7 Framework<br>National, Local and Private Initiative	and **civilians,** boost cross-domain **industry collaboration,** and achieve local and industrial **innovation**. (Taiwan Ministry of Digital Affairs, n.d.)	website, Open Platform for Government Information of the New Taipei City.<br>https://data.ntpc.gov.tw/<br>Taipei City Data Big Platform<br>https://data.taipei/	Living Lab +
**15th) Barcelona (ES)**<br><br>Since 2000:<br>22@Barcelona Project<br>2008Urban Lab<br><br>Since 2011:<br>IT Strategy aligned to EU Horizon Strategy<br>Smart City Strategy<br>Smart City Expo and World Congress<br><br>Since 2015:<br>Barcelona Digital City Plan<br>EU, Local, and Private	D1 and V1: 'To become a **self-sufficient city** of **productive neighborhoods** at **human** speed, within a hyper-**connected zero emissions** metropolitan area' (Olivella, 2012; Ferrer, 2017 p. 73; T. Miao and Phelps, 2019 p. 14)<br>T1: Self-sufficient, hyperconnected, urban lab<br><br>D: Not found<br>T2: **Digital, Sovereing City**<br>V2: not clear, but there is this declaration: Barcelona aspires to evolve the Smart City Agenda towards becoming a **digital sovereign** city, empowering **citizens** to discuss and articulate their own priorities, set direction, and decide upon ethical uses of **technological innovations** with clear **social** impact and **public** return. (Ajuntament Barcelona, 2019 p. 5).	Fields: Economy, Governance, People, Environment<br><br>Goal: Efficiency, Sustainability, QoL<br><br>TA: Connection, Technology<br><br>Others: Innovation, City, Stakeholders<br><br>Digital platforms:<br><br>Aplicaciones.bcn<br>Barcelona in your pocket<br>Decidim Barcelona<br>Governance Model<br>Participa.barcelona<br>Wesolve – Better Together app<br>Open Data BCN	City Lab Barcelona<br><br>Fab City Hub Barcelona<br><br>Fab Lab Barcelona<br><br>Data City Lab<br><br>22@LIVING Lab<br><br>The Healthcare Living Lab Catalonia (HCLLC)<br><br>The Library Living Lab – Barcelona (L3)

**Rank and Initiatives**	**Smart City Definition (D), Type (T), and Vision(V)**	**Terms and Digital Platforms**	**Fab, Living or Urban Labs**
**16th) Toronto (CA)**<br>In 2016:<br>TRBoT created the Smart Cities Working Group<br>In 2017, the Canada Government launched the Smart City Challenge<br>In 2018:<br>Toronto Smart City Challenge Submission Connected Community Smart City Project (?)<br><br>National & Local	D: Is a **city** that improves access to **information** and **data**, to help it become an **economically, socially, and environmentally connected community**. (City of Toronto, 2017).<br><br>T: **Connected Community**<br><br>V: not found	Fields: Economy, People, Environment<br>Goal: Efficiency, Sustainability, QoL<br>TA: Connection, Information<br>Other: City, Data<br>Digital platforms:<br>ConnectTO program<br>The City of Toronto website, the Trip Planner app, The MyWaterToronto Portal,<br>The 311 Toronto App, Cowlines App, Cycle Now app, Garbage Day App, Transit App,<br>Toronto Open Data<br>https://open.toronto.ca/<br>Open Data Master Plan 2018-2022	Future Living Lab<br><br>Google Quayside Sidewalk Toronto<br><br>University of Toronto Living Lab
**17th) Paris (FR)**<br>Since 2010:<br>Urban Lab<br>Since 2015:<br>Paris Smart and Sustainable Plan – Looking ahead to 2020&Beyond	D: A City that develops common services, **applications,** and **goods** that meet the **basic needs** of its **inhabitants** and their quest for **well-being**. It is an **innovative** approach based on a medium and long-term global and systemic vision of the city to **improve the quality of life** by leveraging three	Fields: Economy, People, Environment, Governance, Living<br><br>Goal: Sustainability, QoL<br><br>TA: Connection, Technology	Agrilink<br><br>3E-Paris Periurban Lab<br><br>La Fabrique du Futur

Local and Private Initiative	major catalysts to transform **urban life**: deepening social ties and **creating value**, rethinking **urban infrastructures**, and capitalizing on **technological** revolutions, especially **digital technologies**. (Mairie de Paris, 2015 p. 5)<br>T: **Connected, Open, Sustainable** City<br>V: Our vision of **Smart and Sustainable** Paris is based on three major pillars: the **open city**,, the **connected city** and the **sustainable city**. Each one of these brings value and resources to the comprehensive approach that will help develop the new trajectory of this 21st-century metropolis. **Data** use and **governance** will also influence these three dimensions for the city of tomorrow. (Mairie de Paris, 2015 p. 25)	Other: Open City, Applications, Data<br><br>Digital Platforms to Engage Citizens:<br><br>Budget Participatif<br><br>Data.gouv.fr<br><br>Réinventer Paris<br><br>Open Data Paris https://opendata.paris.fr/	Liberté Living Lab<br><br>Le Rosa Lab – Fabrique de La Ville Creative et Durable<br><br>Universcience Living Lab
**18th) Madrid (ES)**<br>Since 2015: Madrid Intelligence Project (MiNT)<br>Since 2022: Madrid Digital Capital - Transformation Strategy for the	D: Holistic vision of a **city** that applies **ICTs** to improve the **quality of life** and accessibility of its **inhabitants** and ensures **sustainable economic, social and environmental development** in permanent improvement. (MINETAD, 2015 p. 3)<br>In partnership with IBM, in 2015, Madrid Intelligence Project (MiNT) was implemented to improve **urban services** and the **quality of citizens'**	Fields: Economy, People, Environment, Governance, Living, People<br><br>Goal: Efficiency, Sustainability, QoL<br><br>TA: ICTs, Technology<br><br>Other: Open, Inclusive, Digital Transformation	Madrid International Lab<br><br>The Internet of Things Laboratory of the City of Madrid (IoTMADLab)<br><br>Madrid Urban Lab

City of Madrid<br><br>Private, Local and UE	**services,** to make the city more **sustainable** (IBM, 2014).<br>T: An **Open, Inclusive, Digital**, and **Sustainable** City<br>V: a strategy to continue being a city benchmark in the **digital** field, designed for all **people** and **companies** that are part of Madrid, for its neighborhoods and districts, and for the City Council itself, as a key driver of the **digital transformation** of the capital (Ayuntamiento de Madrid, 2022 p. 4-7)	Digital Platforms to Engage Citizens:<br><br>Decide Madrid<br><br>Getafe Participa<br>SmartAppCity<br>Open Data Madrid Portal<br>datos.madrid.es/portal/ site/egob/	Observatorio de La Ciudad<br><br>The Madrid Smart Lab
**19th) Busan (SK)**<br><br>In 2017: won IBM SCC<br><br>Since 2018:<br>Busan Eco Delta City: A National Smart City Project<br><br>Local, Private, National	Not found a local definition, but national.<br>D1: A **platform** to improve the **quality of life** for **citizens**, enhance the **sustainability** of cities, and foster **new industries** by utilizing **innovative technologies** of the **4th Industrial Revolution** era. (South Korea Ministry of Land, Infrastructure and Transport, 2019 p. 5)<br>T: **Digital, Augmented, and Robotic** City (Busan Metropolitan City, n.d.)<br>Definition of the project: Three (**Digital, Augmented, and Robot City**) future city operation platforms, where all **citizens** will benefit from balanced opportunities and **inclusive growth** as a mecca of the **future industry** and improve the **quality of**	Fields: Economy, Environment, Governance, Living, People<br>Goal: Efficiency, Sustainability, QoL<br>TA: Technology<br>Other: Platform, Innovation, Digital, Augmented, Robot, 4th Industrial Revolution, International Leadership<br><br>Digital Platforms to Engage Citizens:<br><br>Busan Eco Delta Smart City Portal<br>www.busan.go.kr/eng/	Busan Innovation Fab Lab<br><br>Busan EDC Village<br><br>Busan's IoT Living Lab<br><br>Busan Network of Living Lab (BNoLL)

	**life** of **citizens,** including **education, culture, safety, and environment** (South Korea Ministry of Land, Infrastructure, and Transport, 2021).<br>V: a place of **innovative** and **international leadership** for future **living** where **nature, people and technology** come together.	ecodelta01<br>Busan Open Data Portal<br>data.busan.go.kr/<br><br>Busan Metropolitan City Big Data Platform<br>bigdata.busan.go.kr/<br>City-Bot	
**20th) Dublin (IE)**<br><br>Since 2012: Intel Labs Europe, Dublin City Council and Trinity College start Sustainable Connected Cities<br><br>In 2014: Won IBM Smart City Challenge<br><br>Since 2016: Smart Dublin Program<br><br>Local and Private Initiatives	D: A **real-time, connected, and data-driven city.** (Smart Dublin, n.d. p. 3)<br><br>T: **Open, Connected, and Engaged** City.<br><br>**Open** to promote **economic development** through **open data**, **open government,** and **open innovation**. **Connected** to drive **efficiencies** through connected **networks**, connected **infrastructure,** and connected **city region**. Engaged to **engage** with **entrepreneurs, citizens, researchers, and business** to solve city region challenges.<br>(Dublin City Council, n.d. p. 2)<br><br>V: To be a world leading **open, connected, and engaged Smart City Region** to **live in, work in and visit**. (Smart Dublin, n.d. p. 6)	Fields: Economy, Governance, Living, People<br><br>Goal: Efficiency, QoL<br><br>TA: Connection, Data, Innovation<br>Other: Real-time, Data Driven, Open, Engage<br><br>Digital Platforms to Engage Citizens:<br>Smart Dublin website<br><br>Dublin Dashboard<br>Smart Dublin Public Trello Board<br>See.Sense<br>Smart Dublin Challenges<br><br>Open Data Dublinlinked<br>data.smartdublin.ie/	I-CHANGE<br>Dublin Living Lab University College Dublin (UCD)<br><br>iSCAPE Dublin Living Lab<br><br>Dublin Coastal City Living Lab

Rank and Initiatives	Smart City Definition (D), Type (T), and Vision(V)	Terms and Digital Platforms	Fab, Living or Urban Labs
**21st) Melbourne (AU)**<br>In 2015:<br>Won IBM Smart City Challenge<br>In 2021:<br>Economic Development Strategy 2031<br><br>Community Engagement Policy<br>Melbourne neighborhood planning framework<br>Local & Private	D: Not found.<br>T: City as a **System** (ARUP, 2010 p. 12). A **City Lab** (City of Melbourne, n.d.)<br>V: To enhance the aspects of our **city** that make us **uniquely** Melbourne, and **intelligently** prepare for the changing needs of the **community**, the **environment,** and the **economy**. (City of Melbourne, n.d.)<br>T2: **Digitally Connected** City, adapting to connectivity as a **knowledge-enabled Smart City**<br>V2: By 2031 the City of Melbourne will be a **global leader** in creating **jobs** and **enterprises** that focus on the needs of the future.<br>City of Melbourne (2021 p. 6)	Fields: Economy, Environment, People<br>Goal: Efficiency, Sustainability, QoL<br>TA: Technology, Connected<br>Other: City, Innovation, Open Data, System<br><br>Digital platforms: Participedia, CityLab<br>FreeWifi, Melbourne Conversations, Neighborhood Portals<br>http://bit.ly/3YgrXoz<br><br>Melbourne Open Data<br>data.melbourne.vic.<br>gov.au/pages/home/	Future Self and Design Living Lab<br><br>Infosys Melbourne Living Lab<br><br>RMIT Healthy Liveable Cities Lab<br><br>Swinburne Living Lab
**22nd) Los Angeles (USA)**<br><br>Since 2020<br>SmartLA 2028 strategy<br><br>Local and Private Initiative	D: Is one that **efficiently** and **ethically** uses secure **technologies, data, &** **resources** to improve **quality of life** and **sustainability** for **residents, businesses, and visitors**.<br><br>T: **Digital, and Connected City**<br><br>V: The City of Los Angeles envisions a high **digital** and **connected city** in 2028.<br>City of Los Angeles (2020 p. 3; p. 5)	Fields: Economy, Environment, Governance, Living, People<br>Goal: Efficiency, Sustainability, QoL<br>TA: Connection, Technology, Data,<br>Other: Stakeholders, Resources<br>Digital platforms: MyLA311, LA Metro Transit app, Vision Zero LA, Open Data Portal,	Urban Movement Lab<br><br>UCLA CityLAB

		Cowlines App, LA Open Data - https://data.lacity.org/	
**23rd) San Francisco (USA)**<br><br>Since 2016:<br>San Francisco Smart City Challenge<br><br>National and Local Project	D: a **framework** for using **data, digital technology**, civic and policy **innovation** to enhance the **quality of life** for **all residents**, making cities more **effective, efficient, equitable and responsive (**City and County of San Francisco, n.d., p. 4)<br>T: **Connected** and **Shared** City.<br>V: to make more **shared choices** available so that **people** of all backgrounds can easily get around without needing to own a car. By promoting shared options, we will ensure that more **people** can access **affordable, safe, clean, and reliable transportation** than ever before. (City and Council of San Francisco, 2016)	Fields: Economy, Environment, Living, Mobility, People<br>Goal: Efficiency, Sustainability, QoL<br>TA: Connected, Technology<br>Other: Framework, Data, Digital, Culture of Sharing, EV, Autonomous Vehicles<br>Digital Platforms to Engage Citizens:<br>San Francisco Smart City website<br>SF311 app, SFWifi, SFPark, Cowlines App, Nextdoor app, OpenDataSF<br>datasf.org/opendata/	Create + Build<br><br>San Francisco Smart City Institute<br><br>The Urban Prototyping Lab
**24th) HK (China)**<br>Since 2018:<br>HK Smart City Blueprint 1.0<br><br>Since 2021:<br>HK Smart City Blueprint 2.0<br><br>Local	D: **People** Centric. It should be built upon the needs of the **people,** and the benefits should be seen and felt by **residents** and **visitors** (HK Innovation and Technology Bureau, 2017 p.2)<br>T: **World-class** smart city.<br>V: Embrace **innovation and technology** to build a **world-famed** Smart HK characterized by strong **economy** and high **quality of life.** (HK Innovation and Technology Bureau, 2017 p.3; 2020 p. 3)	Fields: Economy, Living, People<br>Goal: Efficiency, QoL<br>TA: Technology<br>Other: Innovation<br>Digital Platforms to Engage Citizens:<br>Smart City Dashboard, iMSmart App, Geospatial Lab, MyObservatory, HKSmart City website.<br>Open Data HK<br>https://data.gov.hk/en/	CMASS iSPACE<br><br>HKUST Sustainable Smart Campus, Geospatial Lab

**Rank and Initiatives**	**Smart City Definition (D), Type (T), and Vision(V)**	**Terms and Digital Platforms**	**Fab, Living or Urban Labs**
**25th) Montreal (CA)**<br><br>Since 2014: Montreal Smart and Digital City Strategy<br><br>Local and Private	The Strategy mentioned two definitions according to Ville de Montréal (2015 p. 10) and Open North (2018 p. 40)<br>D1: Effective **integration** of **physical, digital,** and **human** systems in the built **environment** to deliver a **sustainable**, **prosperous,** and **inclusive** future for its **citizens** (BSI, 2014).<br>D2: Systems of **people** interacting with and using flows of energy, materials, services, and financing to catalyze **sustainable economic** development, **resilience,** and high **quality of life**; these flows and interactions become smart through making strategic use of **information** and **communication** (European Innovation Partnership on Smart Cities and Communities, 2013).<br>T: **Smart and Digital City**<br>V: By 2017, Montreal will be the world´s leading smart city.	Fields: Economy, Environment, Governance, Living, People<br><br>Goal: Efficiency, Sustainability, and QoL<br>TA: ICT, Information<br>Other: Digital, System, Integration, Communication, resilience.<br><br>Digital platforms:<br><br>Montreal Smart City website, Je fais Mtl<br><br>Civic Tech Montréal, MTL Connect. Montreal BIXI app,<br><br>Montreal Open Data donnees.montreal.ca/	Living Lab de Montréal<br><br>Cité-ID Liv-ing Lab<br><br>Concordia University’s Creative engAGE Living Lab (ELL)<br><br>Rehabilitation Living Lab in the mall (RehabMaLL)
