# Supplementary material for: Guidelines for a participatory Smart City model to address Amazon’s urban environmental problems
Source: PeerJ Comput Sci. 2023 Dec 12;9:e1694. doi: 10.7717/peerj-cs.1694 (PMC10773765; doi:10.7717/peerj-cs.1694)
Supplement: Supplemental Information 6 [file peerj-cs-09-1694-s006.pdf]

DETALHES DA SOLICITAÇÃO

Numero Protocolo:

2831/2022

Tipo Solicitação:

INICIO

Situação:

RESPONDIDO

Forma Retorno:

Email

Data da Solicitação:

23/05/2022

Previsão Retorno:

12/06/2022 09:13:37

Solicitação Recebida em:

15/02/2023 20:17:26

Porrogado:

NÃO

Porrogado para:

/ /

Solicitação:

Prezado Gestor da PMM, Bom dia. Sou professor Jonas Gomes, da UFAM, estou realizando pesquisa sobre Cidades Inteligentes e com base na Lei de Acesso a Informação, gostaria de saber as seguintes informações. Contexto: Em 2016, o então prefeito de Manaus foi reeleito prometendo para os amazonenses transformar Manaus em uma Cidade Inteligente. Então de 2016 até 2022 (quase seis ano): 1) Dimensão Visão: Qual a

visão e estratégia de longo prazo estabelecida para tornar Manaus uma Cidade Inteligente? 2) Dimensão Visão: onde o cidadão encontra o Planejamento Estratégico ou Programa para alcançar essa visão, a fim de transformar Manaus em Cidade Inteligente? 3) Dimensão Liderança: Quem são as lideranças responsáveis para gerir esse Planejamento/Programa? 4 e 5) Dimensão Orçamento: Qual o orçamento alocado para esse empreendimento? em que DOM foi publicado? 6) Dimensão Incentivos Financeiros: Que incentivos financeiros a PMM está oferecendo para estimular Empresários, Universidades, Start Ups e outras organizações para apoiar o Planejamento/Programa? 7) Dimensão Programas de Apoio: Quais programas de apoio a PMM desenvolveu para encorajar o setor privado a participar? 8) Dimensão Preparação de Talentos: Que programa/plano foi desenvolvido para educar o manauara para adquirir habilidades necessárias para atuar em uma cidade inteligente? 9) Dimensão Participação do Cidadão: Quais iniciativas foram efetivamente realizadas para envolver a população no Planejamento Estratégico/Programa para tornar a cidade inteligente? 10) Dimensão Participação do Cidadão: Como a PMM garantirá o efetivo ganho dos benefícios da Cidade Inteligente para a maioria da população? 11 e 12) Dimensão Ecossistema de Inovação: Quem são os parceiros do ecossistema de inovação local que efetivamente estão envolvidos e quais as responsabilidades deles? 13) Dimensão Políticas Inteligentes: Quais as políticas públicas efetivamente desenvolvidas para tornar Manaus Inteligente? 14) Dimensão Políticas Inteligentes: Quais as ações realizadas para atualizar o ambiente regulatório visando apoiar esse empreendimento? 15) Dimensão Registro Histórico: Quais as boas práticas já registradas? 16) Qual o modelo conceitual de Cidade Inteligente está sendo implementado? 17) Em que espaço do site da PMM, o cidadão tem acesso a essas informações? Muito agradecido pela atenção, Prof. Jonas Gomes Departamento de Engenharia de Produção UFAM

Data Resposta:

15/02/2023

Respondido por:

63322757234

Resposta:

Prezado, O REQUERIMENTO, INFORMAMOS QUE O PROFESSOR JONAS GOMES DA SILVA, COMPARECEU A ESTA SECRETARIA, NO MESMO PERÍODO DE 2022, EM REUNIÃO MARCADA COM O ORDENADOR DE DESPESAS DESTA PASTA ONDE SANOU TODOS OS QUESTIONAMENTOS PESSOALMENTE. DIANTE DO EXPOSTO DEVOLVO OS AUTOS PARA PROVIDÊNCIAS CABIVEIS.

Voltar

© 2020 - Todos os direitos reservados à Prefeitura Municipal de Manaus

Versão: 05.31.21
