# Supplementary material for: Guidelines for a participatory Smart City model to address Amazon’s urban environmental problems
Source: PeerJ Comput Sci. 2023 Dec 12;9:e1694. doi: 10.7717/peerj-cs.1694 (PMC10773765; doi:10.7717/peerj-cs.1694)
Supplement: Supplemental Information 7 [file peerj-cs-09-1694-s007.pdf]

UFAM FACULTY OF TECHNOLOGY – INDUSTRY ENGINEERING DEPARTMENT

VIII QUALITY MANAGEMENT COURSE PUBLIC CHALLENGE - V2

REFERRING TO THE EXERCISE OF CLASSES 59 AND 60 - 5 POINTS

Professor Jonas Gomes da Silva – jgsilva@ufam.edu.br

Study Material: Toyota A3 Report and all tools studied.

Public Challenge: The Faculty of Technology´s Direction opened a public call for proposals to select a Startup composed of students to address environmental problems considered priorities in Manaus, based on a survey involving 1,242 city residents between December 9th, 2021, and December 9th, 2022, whose results pointed out the five main environmental problems that most bothered the respondents:

1st) Increased pollution of streams = 974 respondents (78%)

2nd) Increased garbage accumulation on streets = 763 respondents (61%)

3rd) Insufficient urban afforestation = 612 respondents (49%)

4th) Increased air pollution = 501 respondents (40%)

5th) Increased congestion = 493 respondents (39.7%)

To address this, the FT will make available BRL 150,000 for the winner team to face one of the three main problems mentioned, through the implementation of a basic project in a given community, throughout the year 2024 (January to December, 12 months).

The project will be a pilot test that could later be reapplied in other communities if it presents positive results by the end of 2024.

The BRL 150,000 can be used for the acquisition of consumables materials (material that does not become equity and has less than two years duration: stationery, hygiene, disposables, cartridges, etc.), permanent material (material with more than 2 years duration and that can become equity, such as equipment, furniture, etc.), scholarships (maximum three scholarships with monthly value of BRL 1,500 per student, for 12 months), as well as hiring service providers (Example: printing services, consulting, etc.).

To this end, interested parties must be students enrolled at FT, their Startup must have a maximum of 4 students, must choose the team's name, slogan, choose the community, the problem, analyze the causes, effects, as well as the means to combat them.

Each team must propose an action plan to tackle the problem and point out the expected results, using the Toyota A3 Report (use the model) and quality tools learned throughout the course.

Each project will be publicly defended in front of the auditorium of the Faculty of Technology of UFAM, during classes 59 and 60, with evaluators being students and invited professors.

The evaluation criteria are Project Goal, Problem Analysis, Action Plan, Expected Results. For each criterion, the grade scale will be from 1 (Poor) to 5 (Excellent) with the average grade of student

evaluators (NMA) weighing 1 and the average grade of professors and technicians (NMPT) weighing 2, and the final grade of each team will be:

$$NF = (NMA + 2 \times NMPT) / 3$$

TEAMS FROM THE INDUSTRIAL ENGINEERING COURSE

PRESENTATION: February 15, 2023, from 6pm to 8pm

PROBLEM: INCREASED POLLUTION OF STREAMS
START UP: IGARACLEAN
MEMBERS: Luiz Gustavo, Leander, Rodrigo, Vinícius
SLOGAN: Making your tomorrow cleaner

START UP: 4WATER
MEMBERS: Igor Cássio, João Gabriel, Kelly, Suzana
SLOGAN: Water, if you know how to clean it won't be lacking

START UP: BIOHEALTH
MEMBERS: Claudomar, Deborah, Gabriel, Giovanna
SLOGAN: Providing environmental health

PROBLEM: INCREASED GARBAGE ACCUMULATION ON STREETS
START UP: EARTH FRIENDS
MEMBERS: Igor dos Santos, Gláucia, Paulo, Vandeleia
SLOGAN: I preserve therefore I exist

START UP: SPAZZAM
MEMBERS: Carlos, Fernanda Joana, Gabriela, Jennifer
SLOGAN: Choose to be on the clean side

PROBLEM: INSUFFICIENT URBAN AFFORESTATION
START UP: REBIRTH OF VENUS
MEMBERS: Diego, Edson, Fernanda Lima, Leandra
SLOGAN: Plant to Reborn

START UP: URBANIZE WELL

MEMBERS: Gilmar, Joeline, Julie, Rafael

SLOGAN: Leave your mark on the planet

TEAMS FROM THE CHEMISTRY ENGINEERING COURSE

PRESENTATION: February 16, 2023, from 4pm to 6pm

PROBLEM: INCREASED POLLUTION OF STREAMS

START UP: APÊ

MEMBERS: Christian, Gilcllys, Letícia, Julyene

SLOGAN: Stream for all

PROBLEM: INCREASED GARBAGE ACCUMULATION ON THE STREETS

START UP: ENTULHARTE

MEMBERS: Ana, Laís, Rebeca, Suhaila

SLOGAN: Transforming Garbage into Art, do your part too.

START UP: ECOFLONA

MEMBERS: Camily, Karina, Maria,Vinícius

SLOGAN: The sustainable solution

PROBLEM: INSUFFICIENT URBAN AFFORESTATION

START UP: ARBOX LTDA

MEMBERS: Lucas, Karolyna, Vitória, Susan.

SLOGAN: Making the city + clean

START UP: MANAUS ARBOR (WINNER TEAM)

MEMBERS: Brenda, Cury, Guilherme, Patrícia

SLOGAN: Planting seedlings and harvesting changes.
